# Factors influencing orthopedic nurses’ pain management: A focused ethnography

**DOI:** 10.1080/24740527.2017.1403285

**Published:** 2017-12-19

**Authors:** Kayla J. Denness, Eloise C. J. Carr, Cydnee Seneviratne, Janice M. Rae

**Affiliations:** a Acute Pain Service, Alberta Health Services, South Health Campus, Calgary, Alberta, Canada; b Faculty of Nursing, University of Calgary, Calgary, Alberta, Canada

**Keywords:** nursing, qualitative research, orthopedic surgery, postoperative pain management, opioids, fast-track surgery, acute pain, acute care

## Abstract

**Aim**: The aim of this study was to explore the factors influencing orthopedic surgery nurses’ decisions to administer pro re nata (PRN) opioid analgesia for postoperative pain.

**Background**: Fast-track surgery programs reduce length of stay by identifying and addressing factors causing patients to remain in hospital, including pain (H. Kehlet, Lancet. 2013;381:9878(9878)). The management of acute pain is an important component of quality care for patients after total knee arthroplasty.

**Methods**: The study used a qualitative design of focused ethnography. Ten nurses working on an orthopedic surgery unit at a large urban hospital in western Canada participated in semistructured interviews that used a patient vignette to examine factors that influenced participants’ pain management in the context of fast-track surgery. Interviews were transcribed and analyzed using thematic analysis and constant comparison.

**Findings**: Nurses described a complex clinical environment where the interplay of several factors informed decisions to administer PRN opioid analgesia. The unit’s culture and physical space influenced nurses’ assessments of pain and their decisions whether to treat pain with PRN opioids. Each nurse’s self-concept affected pain management decisions because of perceived importance of pain control and perceived duty to provide analgesics. The subjectivity of pain added another layer of complexity as nurses responded to the patient’s expression of pain from within the milieu of the unit culture and their unique self-concept.

**Conclusion**: Understanding the complexity of factors that influence nurses’ postoperative pain management provides clinical nurses and nursing leaders with directions for future education and research, guided by the goal of continued improvement in pain management in the challenging setting of fast-track surgeries.

## RÉSUMÉ

**But**: Étudier les facteurs qui influencent la décision des infirmières en chirurgie orthopédique d’administrer des analgésiques opiacés pro re nata pour la douleur postopératoire.

**Contexte**: Les programmes de chirurgie accélérée réduisent la durée du séjour en cernant les facteurs qui font en sorte que les patients restent à l’hôpital, dont la douleur, et en y apportant des réponses (Kehlet, 2013). La gestion de la douleur aigue est une composante importante de la qualité des soins pour les patients après une arthroplastie totale du genou.

**Méthodes**: L’étude a eu recours à un devis qualitatif d’ethnographie focalisée. Dix infirmières travaillant dans l’unité de chirurgie orthopédique d’un grand hôpital urbain de l’Ouest du Canada ont participé à des entrevues semi-structurées qui avaient recours à une vignette de patient pour examiner les facteurs influençant la gestion de la douleur dans le contexte d’une chirurgie accélérée. Les entrevues ont été transcrites et analysées au moyen d’une analyse thématique et de la méthode de comparaison constante.

**Résultats**: Les infirmières ont décrit un environnement clinique complexe où l’interaction de plusieurs facteurs était à l’origine de la décision d’administrer un analgésique opiacé pro re nata (prn). La culture de l’unité, de même que l’espace physique, influençait l’évaluation des infirmières et leur décision de traiter la douleur avec des opiacés pro re nata. L’image de soi de chaque infirmière affectait ses décisions en matière de gestion de la douleur en fonction de l’importance qu’elle accordait au contrôle de la douleur et de sa perception de l’obligation de donner des analgésiques. La subjectivité de la douleur a ajouté une autre couche de complexité car les infirmières ont répondu à l’expression de la douleur par le patient dans le contexte de la culture de l’unité et de leur propre image de soi.

**Conclusion**: La compréhension de la complexité des facteurs qui influencent la gestion de la douleur post-opératoire par les infirmières donnent des orientations aux infirmières cliniciennes et aux infirmières-cadres en ce qui concerne l’enseignement et la recherche dans l’avenir, dans le but de constamment améliorer la gestion de la douleur dans le contexte difficile des chirurgies accélérées.

## Introduction

Osteoarthritis (OA) is a painful, debilitating disease characterized by the progressive breakdown of cartilage and bone.^1^ Worldwide prevalence of osteoarthritis is growing; it is projected to reach 130 million people worldwide by 2050, causing severe disability in 40 million.^2^ In Canada in 2010, the prevalence of OA was 4.4 million.^1^ By 2020, OA prevalence in Canada is projected to increase by over 50% to affect 6.8 million Canadians.^1^

The projected increase in OA prevalence predicts a substantial burden on health systems globally. When OA is present in the knees, it can cause severe pain and reduced mobility, and the only effective treatment for severe end-stage OA is joint arthroplasty.^3^ In Canada, acute care hospitalizations following joint arthroplasties are necessary but expensive. In 2012–2013, the average cost of one hospitalization for total knee arthroplasty (TKA) surgery was $10 746, with an average length of stay of 3.2 to 4.2 days.^4^

Most patients who undergo TKA surgery every year experience significant postoperative pain.^5–7^ Improvements in pain management following TKA have been associated with early ambulation, thereby lowering the risk of postoperative complications and reducing length of stay.^8^ The advent of fast-track surgery programs in North America and Europe may reduce length of stay by identifying and addressing factors that cause patients to remain in hospital.^9^ One of these factors is pain, so discharge after TKA may be accelerated by effective postoperative pain management.^9^

### Background

Among health care providers in the hospital setting, nurses are best positioned to assess pain and carry out plans to meet the needs of patients. Several barriers to effective pain management have been identified, including nurses’ beliefs and attitudes regarding pain, formal pain assessment skills, time management, competing demands, patients’ anxiety, attitudes toward analgesics, and feelings of helplessness.^10–12^ These barriers may be reinforced or assuaged by the culture and context in which pain management occurs. Willson^13^ used ethnography to identify factors nurses considered when providing analgesia to patients who had surgery to repair a hip fracture. These factors (time management, shift worked, impact of members of the multidisciplinary team, concerns over opioid analgesics, and communication) are consistent with the findings of other authors who have suggested that the culture of the care environment significantly influences pain management.^13–22^

In the time since Willson’s 2000 study,^13^ acute pain management in orthopedic surgery has undergone a revolution. In particular, the emergence of peripheral nerve blocks for postoperative analgesia has resulted in improved pain scores, reduced opioid requirements, and improved mobility in TKA.^23–27^ In recent clinical practice guidelines, Chou et al.^28^ strongly recommended peripheral nerve blocks as an important component of multimodal analgesia in surgeries such as TKA. However, a peripheral nerve block resolves within the first 36 h postoperatively, necessitating a transition to oral opioid analgesics for management of pain, which is typically severe following TKA.^29^ Little is currently known about how to best manage pain during the transition from a nerve block to oral opioid analgesia.

Nurses play an essential role in the management of acute pain. In hospital, short-acting opioid analgesics are frequently prescribed for patients with acute pain on an as-needed or pro re nata (PRN) basis, with nurses often deciding when to administer the PRN opioid. The emergence of fast-track surgery programs has increased the complexity of nursing tasks and increased the number of patients undergoing TKA, which is likely to have an impact on the culture of the practice environment.^30^ It is therefore necessary to consider the effect that this culture has on nurses’ pain management practices.

## Methods

### Aim

The aim of this study was to identify and explore the question, “What factors do nurses consider when deciding whether to give the first dose of opioid to a patient who has had a nerve block for a total knee arthroplasty?” The research question focuses on nurses on an orthopedic surgery unit within a specific context and aligns with the tenets of focused ethnography.

### Design

A review of the literature revealed that the cultural milieu of a practice setting is highly influential in nurses’ management of pain. Ethnography has traditionally been used to explore various cultures, and it has been used by previous authors to explore complex clinical settings.^12,13^ Focused ethnography evolved from the traditional approach as a method of examining specific issues in defined areas, such as a nursing unit, rather than studying a cultural group as a whole.^31^ Focused ethnography shares a common goal with the traditional approach, aiming to identify shared beliefs that generate behaviors.^32^

The first author (KD) works as a clinical nurse specialist in acute pain management, occasionally consulting on the unit where the study was conducted. Knoblauch^33^ discussed a distinction between *strangeness*, a “situation of unfamiliarity not only with specific situations but also with the general culture” (p. 4), and *alterity*, the state of being different from a particular cultural orientation. This is an important distinction, because Knoblauch^33^ argues that focused ethnographies “may only be undertaken under conditions of alterity rather than strangeness” (p. 4). As a nurse researcher and clinical consultant, it would be impossible for KD to maintain a truly *strange* perspective while studying other nurses. However, alterity applies in this context, because KD’s background is in pain management nursing and not in orthopedic nursing. The intention of traditional ethnography is to describe and understand a culture through a combination of the participant’s *emic* view with the *etic* view of the researcher as an outsider.^34^ Alterity preserves the etic perspective within the focused ethnography methodology, in which a common goal is to explore the emic perspective in very specific situations, activities, and actions.^33^

Though a traditional ethnography typically involves long-term fieldwork coupled with extensive description and interpretation, many focused ethnographers choose a targeted data collection approach.^35^ Several authors have completed focused ethnography studies using semistructured interviews as their sole source of data.^31,36–38^ The first author’s consulting role on the unit provided informal observations of the physical layout of the unit; however, no formal observations of the work of nurses on the unit were conducted.

The specificity of focused ethnography provided an excellent framework with which to explore the research question, which relates to a specific practice in a defined area—the orthopedic surgery nursing unit.

### Sample

The sample was orthopedic surgery nurses involved in the care of TKA patients who received a peripheral nerve block for postoperative analgesia. In the province where the study was conducted, registered nurses (RNs) are self-regulated professionals with a 4-year baccalaureate education and a broad scope of practice, and licensed practical nurses (LPNs) have a 2-year college diploma education. Both professional nursing designations are employed on the orthopedic surgery unit, which opened in 2013 with 36 inpatient beds, including one four-bed ward and 32 single-patient rooms. When data were collected, the unit had approximately 75 nursing staff, including RNs and LPNs. This unit was selected because of the high prevalence of TKA patients who receive nerve blocks for postoperative analgesia. The unit is estimated to treat approximately 600 TKA patients yearly.

### Data collection

Following ethical approval, invitations to participate were disseminated to all nurses on the unit through e-mail and posters on the unit. Nurses were asked to express their interest by contacting the first author by phone or e-mail, at which time they were offered an interview appointment. Interviews were conducted by KD in a private room within the hospital between May and July 2015.

#### Semistructured interviews

To orientate the participant to the topic of interest, a patient vignette was used in each semistructured interview. Semistructured interviews have been frequently used in focused ethnographies to inform a researcher of the meanings that a participant assigns to a particular phenomenon.^31,36,38,39^ The vignette was developed by the authors on the basis of a synthesis of literature, expert consultation, and clinical experience. During the interviews, each participant was asked whether he or she felt that the vignette was authentic and realistic. Each agreed that it was and that he or she had cared for patients who had an experience similar to that of Pat, the vignette patient.

**Table:** 

**Patient Vignette**
Pat, 65, has a history of osteoarthritis in the left knee and is otherwise healthy with no known drug allergies. Pat occasionally takes over-the-counter analgesics for knee pain, including regular strength acetaminophen (last taken 3 days ago) and ibuprofen (last taken 7 days ago). Pat is POD [postoperative day] 1 following a left total knee arthroplasty. You review Pat’s chart and have learned that a spinal anesthetic was given, with an adductor canal block delivered in the postanesthesia care unit at 0945 h following surgery. Pat has an order for oxycodone CR 10 mg po q12 h, and the first dose was given POD 0 at 1400 h with a subsequent dose at 0200 h today. No PRN analgesics have been given. Documented pain scores at 0400 h were 0/10 at rest and 2/10 with movement.
**Assessment 1**
During your initial assessment at 0815 h, Pat reports a pain score of 1/10 at rest and 3/10 with movement. Pat denies sedation, nausea, vomiting, and pruritus. Pat reports mild knee stiffness and has adequate motor power in the left leg. You determine that a sensory block is present to the knee and medial aspect of the lower leg. Please review the medication orders and indicate whether you would administer a PRN opioid at this time and which medication and dose you would administer.
**Assessment 2**
At 0945 h, Pat reports a pain score of 2/10 at rest and 4/10 when repositioning in bed. There is a sensory block to the medial aspect of the lower leg, and Pat has intact cold sensation on the knee. Please review the medication orders and indicate whether you would administer a PRN opioid at this time and which medication and dose you would administer.
**Assessment 3**
At 1130, Pat has participated in an initial assessment with a physiotherapist and was able to complete the expected exercises according to the physiotherapy pathway. Pat is resting in bed and reports a pain score of 4/10 with a maximum pain of 6/10 during physiotherapy. There is a sensory block to the medial aspect of the lower leg, and Pat has intact cold sensation on the knee. Please review the medication orders and indicate whether you would administer an analgesic at this time and which medication and dose you would administer.
**Assessment 4**
Pat sits up in a chair to eat lunch. You are called to the room at 1230, at which time Pat reports 6/10 pain while sitting and 8/10 with movement. Pat has normal cold sensation to the left knee and lower leg. Please review the medication orders and indicate whether you would administer an analgesic at this time and which medication and dose you would administer.
**Assessment 5**
At 1330 h, Pat has refused to ambulate with physiotherapy due to pain. Pat reports 8/10 pain at rest and 10/10 with movement. No sensory block is present in the left leg. Please review the medication orders and indicate whether you would administer an analgesic at this time and which medication and dose you would administer.

The vignette included assessment data for Pat at several times during a day shift, as well as a list of medication orders. The list of medication orders was consistent with the typical multimodal analgesic protocol for patients on the study unit and included scheduled acetaminophen (1000 mg every 6 h), scheduled celecoxib (200 mg every 12 h), a scheduled long-acting opioid (oxycodone CR 10 mg every 12 h), and a short-acting PRN opioid (oxycodone 5–10 mg every 4 h PRN). The participant was asked to indicate at what point he or she would decide to administer a PRN opioid analgesic to Pat. The remainder of the semistructured interview included a discussion of why the participant chose to administer the opioid at that time. The vignette and assessment details were identical for each participant.

#### Ethical considerations

Ethical approval was received from the University Conjoint Health Research Ethics Board (Ethics ID: REB14-2367). To mitigate concerns of coercion to participate relating to KD’s professional role on the unit, the recruitment strategy consisted of e-mail and posters and did not allow for face-to-face interaction with potential participants until they expressed interest. Prior to each scheduled interview, participants were given verbal information about the intent and purpose of the study and were asked to read and sign a consent form. The consent detailed the intent to preserve participants’ confidentiality and the opportunity to withdraw from the study.

### Data analysis

Interviews were recorded and transcribed verbatim. Data analysis was an integration of thematic analysis with constant comparison,^40^ consistent with work of other focused ethnographers.^36,39,41–43^ Thematic analysis is a process of organizing data by identifying and labeling emergent themes.^34^ Roper and Shapira^32^ suggested four basic steps in the thematic analysis of ethnographic data: coding, sorting, generalizing, and memoing. Codes are descriptive labels used to summarize ideas and statements made by participants.^32^ Memos are notations of ideas or insights about the data.^32^ Constant comparison occurs simultaneously with thematic analysis. Data analysis was shared among the coauthors throughout the process. Data saturation became apparent when all transcript codes were a good fit for themes that had previously emerged from the data.

### Rigor

Rigor is an essential part of qualitative work to ensure that research data and analysis are trustworthy.^44,45^ The strength of qualitative inquiry is tied to its credibility, transferability, dependability, and confirmability.^46^ Methodological rigor and trustworthiness were maintained through the use of peer review and debriefing among coauthors, rich description, establishment of an audit trail, and reflexivity. KD maintained a reflexive journal during data collection and analysis, facilitating reflection on how past experiences might be impacting the emerging data.

## Findings

Over a period of 6 weeks, 10 nurses out of approximately 75 responded to the study invitation. Participants represented two professional nursing designations (eight RNs and two LPNs) with between 1 and 3 years of practice on the unit. Years of professional nursing experience were 1–5 years (four participants), 6–10 years (four participants), and greater than 10 years (two participants).

The broad array of thoughtful responses from participants reflects the complexity inherent in this area of practice. The variety of participants’ responses were, as articulated by one participant, “all over the map.”

It was evident that the interplay of several factors contributed to pain management following a nerve block. Three interrelated categories emerged from 263 unique codes: (1) unit culture, (2) nurses’ self-concept, and (3) nurses’ perception of the pain assessment.

### Unit culture

Institutional culture has long been considered an important factor in pain management. Glynn and Ahern^17^ suggested that a nursing unit culture should place a high priority on pain management in order to achieve favorable pain outcomes. Participants described how the culture of a unit can affect their approach to pain management:

P9: “I also do try and ask patients, I mean, it’s not something that’s as promoted as much on this unit as it was on my last unit, asking them what their pain goal would be. And trying to go from there.”

P6 also compared the culture of the unit to a previous workplace where patients would often have a prolonged length of stay, which allowed for more “fine-tuning” of pain medication to ensure safety while providing effective pain control. Describing current practices, P6 found that her approach to pain management had changed, and P3 noted the expectation of a 3-day length of stay:

P6: “Who cares if I load on the drugs for three days? Its three days! … I’m far more likely to medicate on the lower end of a pain scale here.”

P3: “It’s a quick pace up here, right? Like, we’re on three days pathway.”

Patients are not discharged until their pain is under control which, as P10 explained, leads to generous dosing of opioids on the unit:

P10: “I would say we give it like candy on ortho. We’re a lot more liberal than other units for pain. If you’re having pain, we’re going to give you something for it.”

P7 described the push to discharge patients quickly as a function of the larger hospital system, with bed shortages leading to pressure from other departments:

P7: “Let’s get them stabilized, let’s get them out the door. And then once they’re out the door then it’s their own problem and they can deal with the case manager or their family doctor. Then it’s done, it’s over and we get a bed. Then we can get somebody out of PACU [postanesthesia care unit] or out of emerg.”

Pressure to discharge patients affects pain management practices of nurses on the unit and may encourage liberal opioid dosing to keep pain under control as a means to expedite discharge.

### Nurses’ self-concept

A significant factor described explicitly by some participants and implicitly by others was their self-concept as a nurse. Hoeve et al.^47^ reviewed various definitions of the notion of self-concept in nursing and endorsed a definition put forth by Takase et al.^48^ as “information and beliefs that nurses have about their roles, values, and behaviors” (p. 197). Participants described the notion of self-concept through articulation of examples of practice uncertainty and perceived obligation to treat pain. P3 expressed a familiarity with practice uncertainty:

KD: “… do you find that difficult in your practice? When you don’t know?”

P3: “No.”

KD: “No?”

P3: “I don’t think that there’s any real … everyone’s different. Right, like, every patient is different, every nurse is different.”

P3’s response illustrated an acceptance of uncertainty as an inherent part of nursing practice and suggested that confidence can coexist with uncertainty.

Participants spoke about their perceived obligation to provide effective pain management. No participant questioned the need to intervene to manage the vignette patient’s pain, indicating that pain management is an underlying assumption embedded in their practice. The management of pain, in the eyes of the participants, included administering medication and teaching patients about pain and analgesics. When asked to provide an example of a particularly challenging patient, P2 described the management of acute pain in a patient who is sensitive to opioids:

P2: “… do I give them the drugs, or do I not? You have to control their pain somehow.”

This perceived obligation to treat pain was echoed by P3:

P3: “We’ve just taken your knee out and put it back together. You’re not going to get out of here pain free right? But we want to make it as close as possible.”

P3’s above quotation combines the obligation to provide effective pain control (“we want to make it as close as possible”) with the assumption that patients need to be taught about pain management (“you’re not going to get out of here pain free”). Participants also described a perceived duty to persuade the patient to take analgesics when the nurse thought he or she should. P4 described a perceived obligation to provide teaching (“it’s our job”), going so far as to “convince” the patient to take pain medication:

P4: “It just depends on the approach of the nurse, in convincing them. Because they totally don’t know about it. We will have to tell them—it’s our job—that the pain will hit. But we also have to prepare them how to control it, how to manage it.”

Some participants described their use of a preconceived number on the Numeric Rating Scale as their own cutoff for providing an analgesic. P10 termed this preconceived number the *nurse’s number*. This approach was also described by P4, P8, and P10:

P4: “Five is the cutoff for me when I *must* give it.”

P8: “I’d usually still wait until pain is 4–5/10 to give them pain medications.”

P10: “But if they’re moving and their pain’s around a 3 or 4, that’s usually my number where I’m going to give them something.”

It was evident that self-concept is an important factor in pain management for TKA patients who have had a nerve block. The preconceived nurse’s number ties in with another important factor identified by participants—the nurses’ perceptions of their pain assessment.

### Nurses’ perceptions of the pain assessment

Participants explained that decision making in pain management becomes extremely challenging when the unit culture and self-concept combine with the interminable variability of the patient assessment. Patients’ expressions of pain are internalized by nurses who develop a perception of the patient’s subjective pain experience that may differ from the patient’s own perception.

Participants reported different practices when assessing pain in a patient who had a peripheral nerve block. Some participants reported that their assessment of the nerve block helped them to decide when to give an opioid, whereas other participants did not consider the intervention to be an important factor in the pain assessment.

P2: “If they had no pain, it means the block is still in. And if they’re starting to have pain, then the block is leaving, so you want something on board.”

KD: “Do you specifically assess the nerve block when you do your pain assessment? Are you specifically looking for the nerve block, or is it just an overall pain assessment that you do?”

P8: “It’s an overall pain assessment.”

KD: “Do you consider the nerve block in your pain assessment?”

P9: “Umm … not a lot actually. I go more from what the patient tells me they’re feeling.”

Participants described challenges faced when integrating peripheral nerve blocks into pain assessments. P5 suggested that nerve block assessments were omitted because no specific assessment tool exists:

P5: “The hard thing for us is that we don’t have assessment tools to score a nerve block. … ’Cause we monitor the spinal, and we don’t have anywhere on our flowsheets to monitor a block.”

Nurses on this unit are expected to use the Numeric Rating Scale (NRS) when assessing pain in their patients. The 11-point scale is a tool for patients to rate the severity of their pain, where a score of 0 is *no pain* and 10 is the *worst pain imaginable*. Participants described how the use of the NRS factored into their pain management decisions for patients who have had TKA surgery with a nerve block:

P7: “Some people, and they are sitting comfortably, you would never guess, but they’re saying they’re 10/10. You know? You get some that are, you know, sitting there, and they’re saying they’re at a 3. And that’s probably where they’re at. It’s different for everybody. Pain is subjective.”

P8: “Some elderly are really bad at telling the pain scores. So their 2/10 might be other people’s 10/10. So if they are about 2/10, I probably would strongly encourage them to take something.”

P9: “Sometimes they tell me their pain scale is really low, and then they’re wincing or groaning, and I’m going, is that really what you’re feeling right now? It is such a difference from patient to patient, what they deem, what a person thinks is a 5/10 and what another person thinks is a 5/10. It’s very, very different.”

These examples reveal the participants’ tendencies to overlay the NRS with their own subjective interpretations of the patient’s experience.

The pain history of patients was another important factor described by participants. Patients undergo TKA surgery to relieve pain in the joint, and many have lived with pain for an extended period of time prior to surgery. Consideration of pain history is especially relevant for this patient population. Participants attested to altering their pain management decisions based on home use of analgesics and chronic pain conditions:

P4: “People who have been on analgesics at home, I am very cautious with them because their need for a pain medication is higher, I like to medicate them more.”

P4: “And some people, if they have other, um, comorbidities, like fibromyalgia … I like to medicate them more often.”

P6: “You know, if they were someone who had chronic pain issues, I probably would give them something on my very first assessment.”

P6 acknowledged that acute surgical pain may be a different experience for patients who have become accustomed to living with pain:

P6: “For these patients that have had bad knees for ten years, their 4/10 might be a relief from their 8/10 that they’ve been living with at home. So that changes things a little bit.”

The subjective nature of pain implies that careful consideration of individual patient needs is an important piece of effective pain management decision making. A history of chronic pain and a patients’ use of analgesics at home was a factor in participants’ pain management decisions.

## Discussion

The uniting element affecting nurses’ decisions to provide analgesics in this study appears to be the established culture of the unit. The unit culture encourages efficiency, which influences nurses’ self-concept regarding the role they play in pain management, in turn affecting their perceptions of the pain assessment. This discussion explores how the unit culture creates tensions for the nurse managing pain and offers insights to reduce those tensions to improve pain management for patients following TKA.

### Tension between efficiency and patient-centered care

The unit exists within an organization that promotes a patient-centered approach to care. Patient-centered care (PCC) occurs when individual patient needs are taken into account in every decision, rather than the goals of the care provider.^49^ The staff and leadership on this unit have fostered a culture with an emphasis on efficiency, with the aim to discharge patients as soon as possible following surgery.

The tenets of PCC align with the subjectivity of individualized pain experiences and may be at odds with practicing efficiency. Efficiency is achieved on the unit through the use of standards and protocols that apply to patients based on the type of surgery they have had, rather than the needs of the unique individual, such as the 3-day pathway. Due to the pressure to discharge patients quickly, there is a risk that unique needs of individual patients may not be met.

Decision makers in health care organizations are inclined to reduce costs and shorten elective surgery waiting lists whenever possible, so the relative importance of efficiency and a shorter length of stay is not likely to change.^50^ Nurses must adapt in order to provide PCC in this environment. However, the practical applications of PCC are not clear. More work is needed to understand how nurses can provide effective PCC in acute hospital environments where interactions with patients are brief and hurried. Initiatives intended to encourage PCC, such as requiring each employee to introduce him- or herself to patients and explain his or her duties and writing names of assigned nurses and aides on white boards in patient rooms, have the potential to improve accountability, which has been cited as a major barrier to pain management in acute care.^10,12,51,52^ However, whether these initiatives alone will lead to improvements in patient-centered pain management is not yet clear. Patient-centered care has been called an “idealized abstraction” because of the challenges in implementing and evaluating PCC interventions in a clinical setting (p. 533).^53^

Nurses need information about how to provide PCC while maintaining efficiency. Without explicit direction on how to integrate the two concepts on the unit, nurses are left in an unsettling environment where the identified priorities seem to be at odds. It would appear that the best opportunity for identifying and anticipating patients’ unique needs may exist outside of the hospital, when TKA patients first engage with the health system in the weeks or months prior to surgery. This is not to say that nurses who work on the unit in this study are not obligated to practice PCC; rather, they should continue work that is started by others who engage with patients prior to their hospital admission. Communication across the continuum of care may be the best way to identify the unique needs of patients while maintaining efficiency. It may also be beneficial to provide ongoing training to orthopedic surgery nurses with the aim to improve their pain assessments, which would provide further opportunity to identify unique needs of patients.

### Informal leaders

Many participants in this study were able to describe practices associated with effective pain management, such as establishing goals, recognizing the subjective nature of pain, and anticipating pain. However, they did not consistently incorporate these ideas into their own practice. Instead, they described generous opioid dosing, prioritizing the need to discharge patients quickly. If the knowledge of individual nurses is to become embedded in the culture of a unit, it is necessary for nurse colleagues to work together and learn from one another, particularly from informal nurse leaders.

Informal nurse leaders are clinical nurses without a formal leadership title. These informal leaders are sought out by peers for guidance and expertise because they have a reputation for being knowledgeable, confident, and approachable.^54^ Pain resource nurses are unit nurses who have received supplementary pain education and support. They can help to make pain a priority within a nursing unit through their informal leadership.^55^

Formal nursing leaders (including managers, educators, and nurse clinicians) should be mindful of the positive effect that informal nursing leaders and pain resource nurses can offer to the overall culture of the unit and should develop targeted strategies to identify, develop, and support informal leaders. Smart^56^ offered some criteria for identifying informal leaders: They are recognized as leaders by peers; they have a wealth of information gained from experience; and they have the ability to motivate coworkers and bring people together. Informal leaders can help to mitigate barriers by providing support to nursing colleagues on the unit.

Participants in this study identified that a prerequisite of effective pain management is an assessment of the individual needs of their patients, which conflicts with their statements pertaining to the prioritization of a quick discharge. They attempted to find a balance between patient needs and the demands of the unit culture, but at times the nurses’ individual knowledge was suppressed by the unit’s culture, such as the generous dosing of opioids in an effort to help patients meet discharge criteria. Participants described barriers to providing effective pain management and explained practices they have adopted to circumvent some of these barriers, but more work needs to be done by formal leaders to clarify priorities and to support and encourage informal leaders and nurse-to-nurse collaboration.

### Physical barriers

A distinctive feature of the unit is that nearly all of the patient rooms are private, with the exception of one four-bed ward. The unit is decentralized, with several small nurses’ workstations distributed along hallways, rather than a centralized design with a single nurses’ station. The physical structure of the unit encourages nurses to work independently, with little opportunity for spontaneous and informal interactions with colleagues.

Estabrooks et al.^57^ found that nurses relied heavily on peer interactions as a primary source of knowledge, especially when faced with practice uncertainty. Decentralized units have been found to reduce nurses’ fatigue because workstations are more proximal to patient rooms; however nurse-to-nurse interactions are less frequent, reducing the potential impact of informal nurse leaders.^58^ Nurses in decentralized units have reported fewer networking opportunities, less teamwork among staff, and less informal mentoring.^59^

The physical structure of the unit, though not explicitly described by participants, may be acting as a barrier to the incorporation of good pain management practices into the unit’s culture. To circumvent the barrier of the decentralized unit, the unit’s leadership should facilitate other opportunities for teamwork and mentoring and cultivate informal nurse leaders.

### Pain discourse

The NRS is a tool that quantifies pain to aid in pain assessment. It has been suggested that nurses may not accurately record a patient’s report of severe pain if nonverbal cues are incongruent with the reported NRS (e.g., a smiling patient reporting a score of 8 on a 0–10 scale).^60^ In alignment with McCaffery et al.’s^60^ findings, participants in this study did not always take NRS scores at face value. Several participants provided examples of patient behaviors that made them question the accuracy of the reported NRS score. These examples, as well as participants’ use of the nurse’s number, illustrate the importance of the nurse’s perception of the patient’s pain expression in his or her pain management practices.

Effective pain management is a result of the relationship between the patient and nurse.^61^ As Tanner^62^ found, decision making is dramatically influenced by what a nurse brings to the situation. Each nurse has a unique combination of a priori knowledge, personal and professional experience, and self-concept. Each patient has medical and psychosocial attributes that are unique and complex. Pain discourse between the patient and nurse is an amalgamation of a nurse’s perception of and response to the patient’s expression of pain. The nurse’s perception and response is influenced by the culture of the unit and the nurse’s self-concept, illustrating the complex relationship among the categories in this focused ethnography.

### Limitations

The scope of this study was focused on one specific unit in order to examine how the unit’s unique cultural and contextual factors impact upon pain management. The findings of this study outline the perceptions, understandings, beliefs, and practices of nurses on this particular unit. Though the specificity of the study precludes the generalizability of the findings, rich description enables readers to achieve transferability. The complexity of acute pain management makes broad theories difficult to apply in practice, and the lack of generalizability is a necessary consequence of qualitative study of a specific clinical context.

Another potential limitation of this study is the primary use of interviews as a data collection method. Behaviors reported in interviews are not necessarily reflected in actual practice; there is a difference between *saying* and *doing*.^63^ This study may have been strengthened by conducting formal observations of nurses’ actual practices in pain management.

Finally, KD’s role as a consultant on the unit may have influenced the participants’ responses. The necessity of alterity rather than strangeness in focused ethnography makes the researcher’s familiarity with the practice environment inevitable. Efforts were made to avoid coercion during recruitment, and the use of memos, debriefing among the authors, and journaling provided an outlet to discuss these issues, fostering an understanding of the participants’ perspectives in the context of the unit’s culture.

## Conclusion

This focused ethnography provided a detailed account of how complex practice environments influence nurses’ management of acute pain. Most patients who undergo total knee arthroplasty surgery experience significant postoperative pain, which may prolong hospitalization and increase the risk of postoperative complications. In the setting of fast-track orthopedic surgery, nurses’ management of acute postoperative pain is impacted by the established culture of the unit, individual nurses’ self concept, and nurses’ perceptions of their pain assessment. Clinical nurses and nursing leaders should use the factors identified in this study as a foundation for developing targeted strategies to improve the management of acute pain in a hospital environment.
